# Reliability of Assessing Hand Osteoarthritis on Digital Photographs and Associations With Radiographic and Clinical Findings

**DOI:** 10.1002/acr.22225

**Published:** 2014-05-27

**Authors:** Michelle Marshall, Helgi Jonsson, Gudrun P Helgadottir, Elaine Nicholls, Danielle van der Windt, Helen Myers, Krysia Dziedzic

**Affiliations:** 1 Arthritis Research UK Primary Care Centre, Primary Care Sciences, Keele UniversityStaffordshire, UK; 2 Landspitalinn University Hospital and University of IcelandReykjavik, Iceland; 3 University of IcelandReykjavik, Iceland

## Abstract

**Objective:**

To investigate the reliability and construct validity of an atlas for grading hand osteoarthritis (OA) on photographs in a separate younger community-dwelling population than the development cohort.

**Methods:**

Participants were community-dwelling adults (ages ≥50 years) in North Staffordshire, UK with hand pain or hand problems in the last year who attended a research clinic. High-quality photographs were taken in a standardized position. A photographic atlas was used to score hand joints (second and third distal interphalangeal [DIP], second and third proximal interphalangeal [PIP], and first carpometacarpal [CMC] joints) and joint groups (DIP, PIP, and CMC joints) for OA on a 0–3 scale. Hand radiographs were graded for OA using the Kellgren/Lawrence (K/L) grading system. Clinical features (nodes, bony enlargement, and deformity) were determined by physical examination. Associations of photographic hand OA grades with radiographic OA and clinical features were determined to assess construct validity.

**Results:**

In total, 558 participants (mean age 64 years, 62% women) were included in the analyses. Reliability for scoring OA on the photographs was good (mean intrarater intraclass correlation coefficient [ICC] 0.77 and mean interrater ICC 0.71). At the joint level, photographic hand OA grade was positively associated with radiographic OA grade (Spearman's ρ = 0.19–0.57, *P* < 0.001) and the number of clinical features (Spearman's ρ = 0.36–0.59, *P* < 0.001). At the person level, individuals with higher global photographic OA scores had higher summed K/L scores and higher percentages meeting the American College of Rheumatology clinical hand OA criteria.

**Conclusion:**

This photographic scoring system was reliable and a good indicator of hand OA in a separate younger community-dwelling population than the development cohort. This method of data collection offers researchers a feasible alternative to physical examination and radiography.

## INTRODUCTION

Hand osteoarthritis (OA) is a highly prevalent condition affecting many older adults (1). Individuals report significant pain and interference with hand function in their everyday lives and perceive their hand condition to be serious (2). Despite this, compared with OA of the knee, there is limited evidence on the epidemiology of hand OA in different populations.

Currently, both clinical and radiographic criteria have their advocates for use in large epidemiologic studies. The radiographic criteria are frequently used to assess the presence and severity of hand OA, and although they are widely available, the disadvantages include cost, radiation exposure, and availability of trained readers (3,4). Furthermore, radiographic changes develop over a considerable length of time, and thus are often underdiagnosed in the youngest and often most symptomatic group of hand OA patients, who constitute a potential future target group for preventative treatment (5,6). The American College of Rheumatology (ACR) criteria are a recognized method of determining the presence of clinical hand OA by physical examination (7), but among the main disadvantages are the availability of expert examiners and the difficulty of standardizing assessments between multiple observers (8). Hand photography offers the possibility of obtaining clinical data in a standardized way, which, if it can be shown to be reliable and a valid indicator of the severity of hand OA, could offer a simple and cheap alternative, particularly if data need to be collected in large samples and over wide geographic areas (9,10).

Photography of the hands has been used in a few studies to examine, and in some cases to diagnose, hand OA (9,11–13). Early investigations suggested that the photographic method lacked sensitivity (9,14), indicating that photographic assessment often missed the presence of radiographic change, but with improved imaging quality and the development of an atlas for scoring hand photographs, the method has shown promise (10,15). The atlas was developed in a population-based study of older adults (ages ≥69 years) in Reykjavik, Iceland (10). This study found that the reading of hand photographs not only could be standardized with reasonable intra- and interreader reliability, but also that the photographic grade of hand OA was correlated with radiographic OA and clinical hand OA (10,16,17), indicating that grades obtained from hand photography may provide a valid indicator of hand OA severity. However, while this system of diagnosing hand OA has been shown to be useful in elderly Icelanders, its performance in other younger populations, where more individuals are likely to be in the process of developing or have an early form of hand OA, is not known. The reliability and validity of an instrument can vary between settings and populations with different clinical characteristics, and it is therefore important to assess these properties across populations and settings in order to confirm the generalizability of photographic assessment of hand OA (18).

The objectives of this study were to investigate the reliability of the Age, Gene/Environment Susceptibility–Reykjavik (AGES-Reykjavik) atlas for diagnosing hand OA from photographs and to assess its validity as an indicator of OA severity by investigating associations with radiographic and clinical features in a separate younger community-dwelling population.

**Significance & Innovations**

- The Age, Gene/Environment Susceptibility–Reykjavik (AGES-Reykjavik) photographic atlas was shown to be a reliable method of scoring hand osteoarthritis (OA) and was associated with both radiographic OA and clinical features.
- This photographic hand OA atlas offers researchers a feasible alternative method of data collection, which may be of particular use for large population-based studies, for studies covering wide geographic or remote areas worldwide, and for researchers wanting to assess widespread involvement that includes hand OA in addition to OA at other joint sites.
- Diagnosing hand OA from photographic images may be of benefit to clinicians providing remote health care because digital images of hands could be taken and sent to the clinician or an expert for assessment.

## SUBJECTS AND METHODS

### Study participants

The Clinical Assessment Study of the Hand (CASHA) is a prospective observational cohort study, in which all individuals ages ≥50 years from 2 general practice registers in North Staffordshire, UK were invited to participate in a 2-stage postal survey. The general practice register was used as a sampling frame because 97% of the population in the UK is registered with a general practitioner (19). Participants were not required to have consulted about their hand pain or hand problems. Individuals who responded to the questionnaires, consented to further contact, and indicated that they had experienced hand pain or hand problems in the previous 12 months were invited to attend a research clinic at a local rheumatology center. The research clinics consisted of a standardized clinical interview, physical examination, digital photographs of the hands, hand radiographs, and anthropometric measurements (height and weight). Full details of the study design and methods have been previously reported (20). The North Staffordshire Local Research Ethics Committee approved this study (project number 1430) and all participants provided written informed consent.

### Digital hand photography

Posterior photographs of both hands were taken using a digital camera (Olympus Camedia C-4040 Zoom; resolution 2,272 × 1,704 pixels). The camera was placed in a fixed position at a distance of 15 inches above a gridded stand. Positioning for the photographs was standardized. The participants were seated with the shoulder adducted and the elbow at 90°. The hand was pronated and placed on a fixed point on the gridded stand with the forearm, wrist, and fingers in a straight line and the hand resting in a natural position, i.e., with the fingers and thumb not held closely together or spanned.

Grading of the dorsal hand photographs was undertaken by a single observer (HJ) using an established scoring system for diagnosing and grading severity of hand OA (10). Five joints in each hand (second and third distal interphalangeal [DIP], second and third proximal interphalangeal [PIP], and first carpometacarpal [CMC] joints) were examined for the visual presence of hard tissue enlargement, deformity, and nodes. Each joint was given a score on a 0–3 scale, with the assistance of a reference photo collection (10), where 0 = normal with no evidence of OA; 1 = mild, some evidence of OA but not fulfilling the criteria for definite disease; 2 = definite moderate OA; and 3 = severe OA. Joint groups across both hands (DIP, PIP, and CMC joints) were also graded for overall involvement of OA using the same 0–3 scale. Hand OA on the photographs was defined as grade ≥2 for a joint or joint group. A global hand OA score was calculated for each participant from the aggregate of the joint group scores (range 0–9). The reader was blinded to clinical and radiographic data. Intrarater reliability was assessed by the reader (HJ) scoring a random sample of photographs from 56 participants a second time after an interval of 4 weeks. A second experienced observer (GPH), who was blinded to clinical and radiographic data as well as the scores of the first reader, also graded a random sample of photographs from 60 participants to determine interrater reliability.

### Radiographic scoring

Dorsipalmar radiographs of the hands and wrists were taken with separate exposures for each hand according to a standardized protocol (20). A single reader (MM), blinded to all questionnaires, clinical assessment, and photographic data, graded all films for the presence and severity of OA using the Kellgren/Lawrence (K/L) grading system, written description (21). Standardized scoring was completed for the second and third DIP, second and third PIP, and first CMC joints in each hand. A second reader, an academic rheumatologist, graded 50 pairs of hands and interrater reliability was found to be very good for the presence of OA in an individual joint (unweighted mean κ = 0.79; 95% mean percentage agreement).

### Clinical features of OA

At the research clinics, a physical examination undertaken by trained physiotherapists and occupational therapists determined the presence of nodes, hard tissue (bony) enlargement, and deformity in the second and third DIP and second and third PIP joints; enlargement and deformity in the first CMC joint; and swelling in the metacarpophalangeal joints. Participants also reported the frequency of hand pain, aching, and stiffness (no days, few days, some days, most days, or all days), which, along with the presence of clinical features, allowed ACR clinical hand OA criteria to be applied (7). The assessors were not aware of the results of the photographic or radiographic scoring, both of which occurred after the clinical assessment.

### Exclusions

Participants were excluded from the analyses if they did not have hand radiographs or digital photographs, or if general practice or local rheumatology medical records or a musculoskeletal radiologist identified them as having inflammatory arthritis (rheumatoid or psoriatic arthritis). Additionally, individuals were excluded if there was an indication of possible inflammatory arthritis or other serious pathology (scleroderma, neuropathic changes, or severe contracture) on the digital hand photographs, as determined by a consultant rheumatologist (HJ).

### Statistical analysis

Statistical analysis was performed using SPSS for Windows, version 14.0. All tests were 2- tailed and a *P* value of 0.05 was considered statistically significant.

The reliability of scoring hand OA from photographs was assessed using intraclass correlation coefficients (ICCs) calculated for absolute agreement using a 2-way random-effects model for single measures for the 10 hand joints and the 3 joint groups for intra- and interrater reliability. An ICC of 0.70 was considered to indicate good reliability (22).

**Figure 1:**
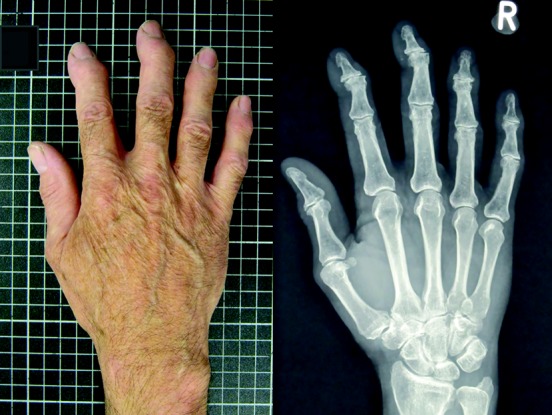
In this study, the associations of photographic hand osteoarthritis (OA) with radiographic OA and clinical features were examined to explore the construct validity of hand photography as an indicator of hand OA. An example of a hand photograph and its corresponding radiographic image is shown.

The associations of photographic hand OA with radiographic OA and clinical features were examined to explore the construct validity of hand photography as an indicator of hand OA (example in Figure 1). For each hand joint and joint group, the frequency of 1) mild (K/L score of 2) and moderate to severe (K/L score ≥3) radiographic OA and 2) the number of clinical features present on the hand examination were determined for each photographic hand OA grade (range 0–3). The radiographic grade and numbers of clinical features for a joint group were determined by the highest radiographic grade and greatest number of clinical features that were present in any of the joints within a group, respectively. Spearman's rank correlation coefficients were calculated to assess the strength and statistical significance of associations of photographic hand OA score with radiographic OA and clinical features at the joint and joint group level. Additionally, global hand OA scores (range 0–9) were compared at the person level using descriptive statistics to 1) summed K/L radiographic scores for all 10 hand joints divided into quartiles and 2) the presence of clinical hand OA according to the ACR criteria (where hand pain was present on most or all days) (7), relaxed ACR criteria (where hand pain was present on some, most, or all days), and the clinical features of hand OA using the ACR criteria (not including the presence of hand pain).

## RESULTS

### Study population

Following exclusions for the absence of hand radiographs (n = 4) or digital photographs (n = 22), definite inflammatory arthritis (n = 28) and possible inflammatory arthritis (n = 8), or other serious pathologies (scleroderma [n = 1], neuropathic changes [n = 1], and severe hand contracture [n = 1]), data from 558 participants were included in the analyses. The descriptive characteristics of the study population are shown in Table 1.

**Table 1:** Descriptive characteristics of the study participants*

	All participants (n = 558)
Women	61.6 (344)
Age range, years	51–91
Age, mean ± SD years	64.2 ± 8.2
BMI, mean ± SD kg/m^2^	28.2 ± 4.8
Attended higher education	16.2 (89)
Manual occupational class	52.3 (274)
Right-handed	90.8 (504)
Hand pain or problems in the last month	85.8 (479)
Thumb pain during activity in the last month	53.0 (296)
Duration of hand symptoms	
<1 year	10.3 (53)
1–5 years	42.3 (218)
>5–10 years	22.5 (116)
>10 years	24.9 (128)
Clinical hand OA	
ACR criteria	29.6 (165)
Relaxed ACR criteria†	51.0 (284)
Radiographic OA	
K/L score ≥2 in ≥1 joint	76.5 (427)
K/L score ≥3 in ≥1 joint	35.8 (200)

* Values are the percentage (number) unless indicated otherwise. BMI = body mass index; OA = osteoarthritis; ACR = American College of Rheumatology; K/L = Kellgren/Lawrence grading system.

† Relaxed ACR criteria are when there is pain on some, most, or all days rather than most days or all days in the ACR criteria.

The frequency of each grade of hand OA and the prevalence of photographic hand OA (grade ≥2) in this study population are shown in Supplementary Table 1 (available in the online version of this article at http://onlinelibrary.wiley.com/doi/10.1002/acr.22225/abstract). The highest prevalence of OA as determined on the digital hand photographs was in the DIP joints on each hand, followed by the first CMC and PIP joints. The same pattern of involvement was seen for the overall joint groups.

### Reliability

Overall, the reliability of grading digital hand photographs on an ordinal scale (range 0–3) was found to be good; the mean ICCs for the 10 individual joints were 0.77 for intrarater reliability and 0.71 for interrater reliability (Table 2). For each joint, the ICCs for interrater reliability tended to be slightly lower than those for intrarater reliability, except for the left second PIP and right first CMC joints, where intrarater reliability was lower.

**Table 2:** Intrarater and interrater reliability for scoring of hand osteoarthritis on a 0–3 scale on digital hand photographs by joint and joint group*

	Intrarater ICC	Interrater ICC
Right		
DIP3	0.87	0.77
DIP2	0.85	0.82
PIP3	0.89	0.82
PIP2	0.74	0.61
CMC1	0.45	0.73
Left		
DIP3	0.91	0.82
DIP2	0.79	0.77
PIP3	0.88	0.64
PIP2	0.36	0.52
CMC1	0.98	0.55
Joint group		
DIP joints	0.83	0.71
PIP joints	0.83	0.77
CMC1 joints	0.93	0.72

* Intraclass correlation coefficients (ICCs) were calculated for absolute agreement using a 2-way random-effects model for single measures. DIP3 = third distal interphalangeal joint; DIP2 = second DIP joint; PIP3 = third proximal interphalangeal joint; PIP2 = second PIP joint; CMC1 = first carpometacarpal joint.

### Associations with radiographic and clinical features

**Figure 2:**
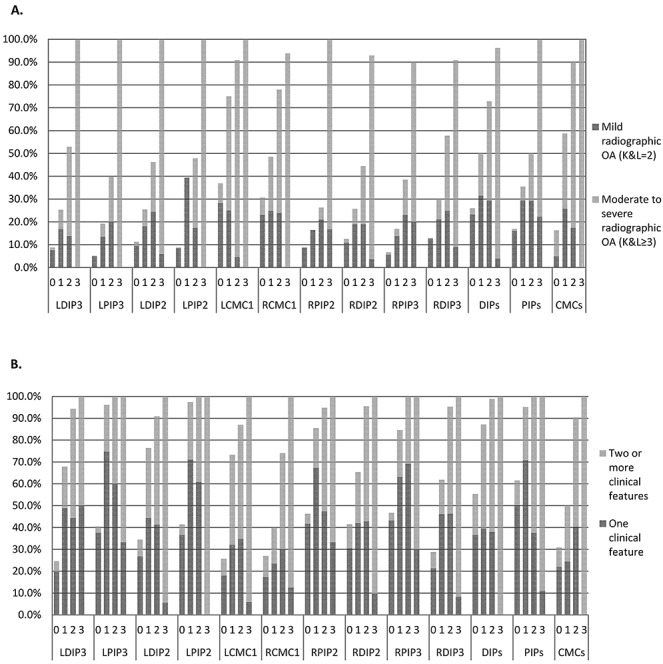
Photographic hand osteoarthritis (OA) grades and the frequency of radiographic OA and clinical features. **A,** For each joint and joint group, the percentage of individuals with radiographic OA increased from grade 0 through to grade 3 of photographic hand OA scores. **B,** For each joint and joint group, the percentage of individuals with ≥1 clinical features as determined on the hand examination increased with photographic grade of hand OA. LDIP3 = left third distal interphalangeal joint; LPIP3 = left third proximal interphalangeal joint; LDIP2 = left second DIP joint; LPIP2 = left second PIP joint; LCMC1 = left first carpometacarpal joint; RCMC1 = right first CMC joint; RPIP2 = right second PIP joint; RDIP2 = right second DIP joint; RPIP3 = right third PIP joint; RDIP3 = right third DIP joint; K&L = Kellgren/Lawrence grade.

For each joint and joint group, the percentage of individuals with radiographic OA increased from grade 0 through to grade 3 of photographic hand OA scores (Figure 2). Of the hand joints and joint groups classed as having photographic grade 3 hand OA, >90% had radiographic OA, the majority of which was moderate to severe OA (K/L score ≥3). However, for those categorized as having photographic grade 2, the amount of radiographic OA present in the joints and joint groups varied greatly from 26–91%. Similarly, for each joint and joint group, the percentage of individuals with ≥1 clinical features as determined on the hand examination increased with photographic grade of hand OA (Figure 2). In hand joints and joint groups categorized with photographic grade 2, >74% had ≥1 clinical features, and in those with photographic grade 3, all (100%) had ≥1 features. Statistically significant associations were found for each joint and each joint group between photographic hand OA score and 1) K/L score (range 0–4) and 2) number of clinical features present (range 0–3) (Table 3).

**Table 3:** Associations of OA on digital hand photographs and radiographic OA and the number of clinical features by joint and joint group*

	Photographic hand OA (range 0–3) and K/L radiographic OA (range 0–4)	Photographic hand OA (range 0–3) and number of clinical features (range 0–3)
Right		
DIP3	0.47 (*P* < 0.001)	0.59 (*P* < 0.001)
DIP2	0.45 (*P* < 0.001)	0.58 (*P* < 0.001)
PIP3	0.32 (*P* < 0.001)	0.41 (*P* < 0.001)
PIP2	0.19 (*P* < 0.001)	0.36 (*P* < 0.001)
CMC1	0.38 (*P* < 0.001)	0.36 (*P* < 0.001)
Left		
DIP3	0.40 (*P* < 0.001)	0.59 (*P* < 0.001)
DIP2	0.42 (*P* < 0.001)	0.59 (*P* < 0.001)
PIP3	0.27 (*P* < 0.001)	0.43 (*P* < 0.001)
PIP2	0.37 (*P* < 0.001)	0.40 (*P* < 0.001)
CMC1	0.57 (*P* < 0.001)	0.51 (*P* < 0.001)
Joint group		
DIP joints	0.50 (*P* < 0.001)	0.54 (*P* < 0.001)
PIP joints	0.29 (*P* < 0.001)	0.38 (*P* < 0.001)
CMC1 joints	0.47 (*P* < 0.001)	0.44 (*P* < 0.001)

* Values are the Spearman's rho. OA = osteoarthritis; K/L = Kellgren/Lawrence; DIP3 = third distal interphalangeal joint; DIP2 = second DIP joint; PIP3 = third proximal interphalangeal joint; PIP2 = second PIP joint; CMC1 = first carpometacarpal joint.

**Figure 3:**
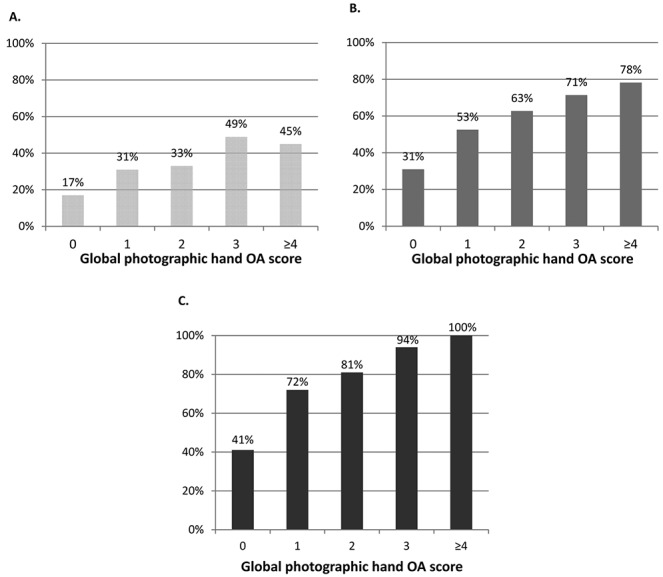
Global photographic hand osteoarthritis (OA) scores were compared with clinical OA at the person level. A higher percentage of those classified as having American College of Rheumatology (ACR) clinical hand OA were represented in those with grades 3 and ≥4 global hand OA scores (**A**). When using the relaxed ACR criteria (where hand pain was reported on some days or more in the last month rather than on most days or more) (**B**) and the ACR clinical hand OA features excluding the hand pain question (**C**), the percentage of individuals meeting the criteria increased as grade of global hand OA increased.

Global photographic hand OA scores (range 0–9), truncated into 5 categories (0, 1, 2, 3, and ≥4) given the small number of individuals with higher grades, were compared to quartiles of radiographic summed K/L scores for the 10 hand joints (0, 1–4, 5–9, and ≥10). Higher K/L summed scores were seen more often in those with higher global hand OA scores (Table 4). Global photographic hand OA scores were also compared with clinical OA at the person level. A higher percentage of those classified as having ACR clinical hand OA were represented in those with grades 3 and ≥4 global hand OA scores (Figure 3). In addition, when using the relaxed ACR criteria (where hand pain was reported on few days or more in the last month rather than on most days or more) and the clinical features of hand OA using the ACR criteria excluding the hand pain question, the percentage of individuals meeting the criteria increased as the grade of global hand OA increased (Figure 3).

**Table 4:** Mean and frequency of summed radiographic scores for different grades of global photographic hand OA*

Global photographic hand OA score (range 0–9)	Summed K/L score (range 0–40), mean ± SD	Summed K/L radiographic score (range 0–40), % (no.)			

		0 (n = 132)	1–4 (n = 149)	5–9 (n = 130)	≥10 (n = 120)
0 (n = 226)	2.9 ± 3.5	39.4 (89)	35.8 (81)	19.0 (43)	5.8 (13)
1 (n = 100)	5.0 ± 4.3	19.0 (19)	37.0 (37)	29.0 (29)	15.0 (15)
2 (n = 82)	6.1 ± 5.2	20.7 (17)	22.0 (18)	37.8 (31)	19.5 (16)
3 (n = 48)	8.7 ± 7.1	14.6 (7)	18.8 (9)	27.1 (13)	39.5 (19)
≥4 (n = 75)	16.6 ± 8.8	0	5.3 (4)	18.7 (14)	76.0 (57)

* Percentages are in rows and show the proportion of summed radiographic quartiles for each global photographic hand OA grade. OA = osteoarthritis; K/L = Kellgren/Lawrence.

## DISCUSSION

This study investigated the reliability of a published atlas for grading the presence of hand OA on high-quality digital photographs in a separate younger population from that in which it was developed. We found the photographic scoring system for hand OA to be reliable for scoring both individual joints and overall joint groups, in terms of both inter- and intrarater reliability. We found photographic hand OA scores to be associated with both the presence and severity of radiographic OA and clinical features of hand OA, confirming the construct validity of the atlas.

Reproducibility of scoring OA on hand photographs has previously been examined (10,13,14). In the same population as the current study, Nicholls et al (14) found only fair agreement (κ = 0.34–0.45) for interrater reliability and moderate agreement (κ = 0.46–0.57) for intrarater reliability. However, some of the raters in this pilot study were inexperienced in assessing clinical hand OA features, and the presence of nodes, bony enlargement, and deformity was assessed individually rather than globally. In contrast, Stankovich et al (13) found excellent intrarater reliability, with ICCs >0.94 for the presence of nodes in the DIP joint group, and Jonsson et al (10) found good reproducibility using a global assessment of features, with intrarater ICCs of 0.81–0.95 and interrater ICCs of 0.78–0.89. In the current study, the intra- and interrater reliability was slightly lower than that reported in the AGES-Reykjavik study. However, this was probably due to using ICCs for single measures, which obtained estimates of reliability for a single rater, and results in values that are lower than those obtained for average-measures ICCs. The single-measures ICC is considered to be more appropriate to estimate intra- and interrater reliability for future studies that will not repeat the same degree of testing with multiple raters on multiple occasions (23).

A number of previous studies have tested the diagnostic accuracy of examining OA features on hand photographs using radiography as the reference standard for hand OA, and have reported inconsistent findings (9–11). It is questionable whether radiography is an adequate reference standard for hand OA, given the known discordance between clinical and radiographic features of OA, with radiographic definitions of OA producing higher prevalence estimates compared with clinical definitions (24,25). Therefore, in the current study, we decided to focus on construct validity by investigating associations of photographic hand OA scores with radiographic OA and clinical features of hand OA. In our study population, those with at least moderate (grade 2) photographic hand OA in each joint or joint group displayed a stronger association with clinical features than with radiographic OA. This was also seen in a previous study by Jonsson et al (10) in the AGES- Reykjavik Study. The strength of associations obtained for construct validity between photographic hand OA and clinical hand OA (Spearman's ρ = 0.36–0.59) was greater than that obtained in a previous study (26) examining correlations of clinical OA with radiographic changes (r = 0.18–0.52), which was comparable to the correlations in the present study between photographic and radiographic hand OA (Spearman's ρ = 0.19–0.57). Stronger associations were expected because we assessed similar constructs when comparing clinical hand OA features determined by a physical examination with the same features assessed visually on digital photographic images. Clinical and radiographic features of OA may represent slightly different presentations of OA that may not always coexist or that occur at slightly different time points, particularly in early OA. Despite the weaker associations, radiographic OA was present in almost all hand joint and joint groups with severe (grade 3) photographic hand OA.

Lower photographic hand OA grades showed a wider range of K/L scores compared with higher photographic grades. This might have occurred for several reasons. First, in early OA, some individuals may present with clinical features and some with mild radiographic OA, and it is possible that at this early stage, clinical features and radiographic changes may not always coexist in the same joint. However, once the disease has become more established, individual hand joints are more likely to be affected by both clinical features of OA and radiographic changes. Second, it is possible that there is a time lag between clinical features of hand OA being detected through a hand examination and being able to clearly observe them on a photographic image, thereby leading to some disparity between photographic and radiographic OA, particularly in early OA. Despite this, trends in the data showed that as photographic hand OA grade increased, there were corresponding increases in the radiographic OA scores.

The assessment of OA on digital hand photographs offers researchers a potential alternative for collecting clinical hand OA data. It has the advantage of being a simple and cheap method that can be undertaken by a single centralized researcher trained in the photographic protocol. This method may be of particular benefit if the data collection is taking place over a wide geographic area or if recruitment is occurring in remote areas, and therefore may especially be of use in studies wishing to examine the effects of race and ethnic origin on the prevalence of OA, which to date have shown some interesting disparities (27–29). Training different individuals to carry out a standardized photographic protocol to capture images would be easier than trying to standardize multiple observers determining the presence of clinical features on a hand examination. A photographic method of assessing and diagnosing hand OA also has potential for use in studies of OA at other joint sites, such as the knee, hip, or foot. Researchers may be interested in assessing more widespread involvement that includes nodal hand OA, but time, cost, radiation exposure, and availability of expert examiners limit the possibility of radiography or standardized clinical assessments of the hand. Additionally, the method of diagnosing hand OA from photographic images may be of benefit to clinicians providing remote health care, particularly those providing consultations at a distance from their patients. Photographs of hands could be taken and sent to the clinician or an expert for their assessment. Photographic images also offer the benefit of providing a permanent record of an individual's hands at a specific time point and can be revisited for other features at a later date, if necessary, or compared to future images. The global scoring of joint groups, which can also provide an overall hand OA score, showed good reliability and construct validity with radiographic summed score and the ACR criteria for clinical hand OA. This was particularly the case when global photographic hand OA scores were compared to the ACR clinical hand OA features without the inclusion of the pain question.

There are a few limitations that should be considered when interpreting the results from these analyses. The oblique positioning of the thumb when the hands were pronated and placed palms down made the first CMC joint harder to assess and grade for photographic hand OA. This may explain the lower interrater reliability for the left first CMC joint and lower intrarater reliability for the right first CMC joint; however, the inter- and intrarater reliability for the overall CMC joint group was good. It is possible that additional views with the hands supinated may be useful to help capture features in this joint. All individuals in this population cohort had hand pain or hand problems in the previous 12 months. While individuals with inflammatory arthritis and scleroderma were excluded, other hand conditions (such as carpal tunnel syndrome, Dupuytren's contracture, and trigger finger) may have been present, with or without the co-occurrence of hand OA. When there were indications of other conditions visible on the photographs that would have affected the photographic grading, as determined by the main assessor (HJ) who is an experienced rheumatologist, these individuals were excluded from the analyses. Therefore, we believe that the presence of these other hand conditions did not strongly influence the findings of this study. Additionally, nodes and hard tissue (bony) enlargement were assessed as separate features in the interphalangeal joints; however, we acknowledge that nodes are a form of hard tissue enlargement. At times, it may be difficult to differentiate between the 2 features, and in some instances, nodes may have been categorized as bony enlargement or vice versa (30). The analysis of clinical features in this study was based on the total number of clinical features present; any misclassification between the two was unlikely to have altered the findings of this analysis. In addition, for the assessment of the ACR hand OA criteria, the presence of either bony enlargement or nodes was used to represent hard tissue enlargement, required to be present in ≥2 of the 10 selected joints and ≥2 DIP joints.

The AGES-Reykjavik photographic scoring system for hand OA has been shown to be reliable and a valid indicator of hand OA as assessed by radiographic and clinical features, and its use in the current study confirmed the adequate properties of this scoring system in a separate, younger community-dwelling population of individuals with hand pain or problems. This method of data collection offers researchers a feasible alternative to physical examination and may be of particular use in large studies and studies covering wide geographic or remote areas.
