# Supplementary material for: Reliability of Assessing Hand Osteoarthritis on Digital Photographs and Associations With Radiographic and Clinical Findings
Source: Arthritis Care Res (Hoboken). 2014 May 27;66(6):828–36. doi: 10.1002/acr.22225 (PMC4153954; doi:10.1002/acr.22225)
Supplement: Supplementary file 1 — Supplementary Table [file acr0066-0828-SD1.docx]

**Supplementary Table 1. Frequencies of grades of OA and the prevalence of hand OA as identified from digital hand photographs by joint and joint group**

|  |  | **Grade 0 % (no)** | **Grade 1 % (no)** | **Grade 2 % (no)** | **Grade 3 % (no)** | **Prevalence of Photographic OA^†^ % (no)** |
| --- | --- | --- | --- | --- | --- | --- |
| **Right** | DIP3 | 63.7% (347) | 14.1% (77) | 20.0% (109) | 2.2% (12) | 22.2% (121) |
|  | DIP2 | 46.8% (256) | 19.6% (107) | 28.0% (153) | 5.7% (31) | 33.6% (184) |
|  | PIP3 | 81.2% (437) | 12.1% (65) | 4.8% (26) | 1.9% (10) | 6.7% (36) |
|  | PIP2 | 85.3% (466) | 10.1% (55) | 3.5% (19) | 1.1% (6) | 4.6% (25) |
|  | 1CMC | 66.7% (329) | 19.9% (98) | 10.1% (50) | 3.2% (16) | 13.3% (66) |
| **Left** | DIP3 | 68.3% (379) | 15.1% (84) | 15.9% (88) | 0.7% (4) | 16.6% (92) |
|  | DIP2 | 56.1% (313) | 19.0% (106) | 21.7% (121) | 3.2% (18) | 24.9 (139) |
|  | PIP3 | 87.2% (476) | 9.5% (52) | 2.7% (15) | 0.5% (3) | 3.3% (18) |
|  | PIP2 | 88.9% (495) | 6.8% (38) | 4.1% (23) | 0.2% (1) | 4.3% (24) |
|  | 1CMC | 81.3% (417) | 10.9% (56) | 4.5% (23) | 3.3% (17) | 7.8% (40) |
| **Joint group** | DIPs | 51.9% (287) | 26.6% (147) | 16.6% (92) | 4.9% (27) | 21.5% (119) |
|  | PIPs | 79.1% (436) | 14.9% (82) | 4.4% (24) | 1.6% (9) | 6.0% (33) |
|  | 1CMCs | 68.9% (373) | 18.1% (98) | 9.6% (52) | 3.3% (18) | 12.9% (70) |

*^†^ OA was defined as ≥grade 2 in a joint or joint group,* *DIP, Distal Interphalangeal joint; PIP, Proximal Interphalangeal joint; 1CMC, 1^st^ Carpometacarpal joint.*
